# A Social Worker-Led Primary Palliative Care Model for Hospitalized Patients Admitted to the Hospital Medicine Service

**DOI:** 10.1089/pmr.2020.0093

**Published:** 2020-10-22

**Authors:** Keisha Berglund, Emily Chai, Jaison Moreno, Maria Reyna, Laura P. Gelfman

**Affiliations:** ^1^ Department of Social Work Services, Mount Sinai Medical Center, New York, New York, USA.; ^2^ Brookdale Department of Geriatrics and Palliative Medicine, Department of Medicine, Icahn School of Medicine at Mount Sinai, New York, New York, USA.; ^3^ Division of Hospital Medicine, Department of Medicine, Icahn School of Medicine at Mount Sinai, New York, New York, USA.; ^4^ Geriatric Research Education and Clinical Center, James J. Peters VA Medical Center, Bronx, New York, USA.

**Keywords:** palliative care, quality and outcomes, social worker

## Abstract

***Objective:*** To increase earlier access to palliative care, and in turn increase documented goals of care and appropriate hospice referrals for seriously ill patients admitted to hospital medicine.

***Background:*** Due to the growing number of patients with serious illness and the specialty palliative care workforce shortage, innovative primary palliative care models are essential to meet this population's needs.

***Methods:*** Patients with serious illness admitted to hospital medicine at a quaternary urban academic medical center in New York City and received an embedded palliative care social worker consultation in 2017. We used univariate analyses of sociodemographic, clinical, and utilization data to describe the sample.

***Results:*** Overall, 232 patients received a primary palliative care consultation (mean age of 69 years, 44.8% female, 34% white, median Karnofsky Performance Status of 40%), and 159 (69%) had capacity to participate in a goals-of -are conversation. Referrals were from palliative care solid tumor oncology trigger program (113 [49%]), specialty palliative care consultation team (42[18%]), and hospital medicine (34[14.6%]). Before the consultation, 10(4.3%) had documented goals of care and 207 (89%) did after the consultation. The percentage of those referred to hospice was 24.1%. Of those transferred to specialty palliative care consultation service, nearly half required symptom management.

***Discussion:*** Patients who received a primary palliative care consultation were seen earlier in their illness trajectory, based on their higher functional impairment, and the majority had capacity to participate in goals-of-care discussions, compared with those who were seen by specialty palliative care. The consultation increased goals-of-care documentation and the hospice referral rate was comparable with that of the specialty palliative consultation team.

## Introduction

Over the last decade, hospital-based specialty palliative care has been one of the fastest growing fields in medicine.^1^ The goal of palliative care is to improve the quality of life (QOL) for patients living with serious illness and their families, and it is appropriate at any age or any stage of a serious illness. In addition, it can be provided along with curative treatment.^2,3^ Specialty palliative care is provided by a specialty-trained interdisciplinary team of physicians, nurse practitioners, social workers, chaplains, as well as pharmacists, art therapists, and massage therapists, who work together with other doctors to provide an extra layer of support. Typically, this interdisciplinary team works with primary clinicians to consult or comanage the palliative care needs of patients with serious illness and their caregivers. A recent systematic review and meta-analysis of specialty palliative care interventions suggest that a palliative approach is associated with improved patient QOL, reduced symptom burden, and improved caregiver outcomes.^4^ Yet, due to specialist palliative care clinician workforce shortages^5,6^ and the growing number of patients with serious illness with palliative care needs (pain and symptom management, clarification of goals of care, psychosocial support),^7^ there is a critical need to develop innovative primary palliative care models^3,8^ with existing resources.

In contrast to specialty palliative care, primary palliative care refers to the fundamental palliative care provided by all clinicians, regardless of discipline or specialty.^8^ Specifically, primary palliative care includes basic management of symptoms, including pain, shortness of breath, nausea, depression, and anxiety, in addition to the facilitation of goals-of-care discussions in the context of the serious illness.^8^ In contrast, specialty palliative care focuses on management of complicated symptoms, such as refractory pain, depression, anxiety, and existential distress, as well as discussions about goals of care when there is conflict between providers or within the family.^8^ By training all clinicians in primary palliative care, patients with serious illness and their families will have increased access to palliative care. Hospital medicine physicians, or inpatient physicians who work exclusively in a hospital, receive little to no primary palliative care training, yet they care for a population of patients with serious illnesses, and in turn patients who have unmet palliative care needs.^9^

**FIG. 1.**
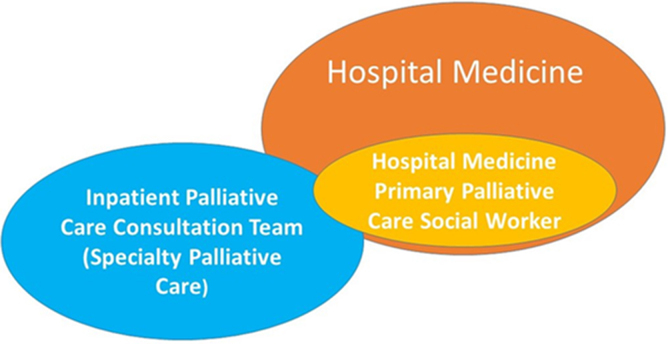
Social worker-led primary palliative care embedded in hospital medicine. This figure demonstrates the intersection and overlap between the Hospital Medicine service, the Inpatient Palliative Care Consultation Team, and the Hospital Medicine Primary Palliative Care Team. Patients who received a consultation by the Hospital Medicine primary palliative social worker (light orange oval) represent a subset of patients admitted to the Hospital Medicine service (dark orange oval), of which a small subset receives a specialty palliative care consult by the Inpatient Palliative Care Consultation Team (overlap of light orange and blue ovals).

There is a growing need for hospital medicine physicians to access palliative care for their patients.^10–12^ However, hospital-based palliative care resources are too limited to ensure that these patients receive palliative care.^13^ Therefore, to meet the palliative care needs of these seriously ill patients at Mount Sinai Hospital, a 1144-bed quaternary-care teaching facility located in New York City, we initiated an innovative social worker-led primary palliative care model embedded within the hospital medicine service (Fig. 1). This primary palliative care program set out (1) to identify opportunities to engage patients facing serious illness and their families earlier in their disease course, (2) to increase the rate of upstream goals-of-care conversations occurring in collaboration with hospital medicine, and (3) in turn to increase alignment of patient preferences and medical treatments received. The scope of practice of the social worker included assisting hospital medicine teams with discussions about illness understanding, prognosis, goals of care, advance care planning (ACP), and discharge planning. In this article, first, we describe the development of social worker-led primary palliative care clinical model, and second, we describe the patient population served by the program, rates of documented goals-of-care discussions, and the health care utilization of this population.

## Methods

### Design

#### Clinical model development

##### Primary palliative care training of the hospital medicine social worker

First, we identified an experienced social worker known to the hospital medicine service to lead the embedded hospital medicine primary palliative care model. The palliative care training for this social worker included the following: (1) completion of Center to Advance Palliative Care communication skills training modules (CAPC.org) before hands-on clinical care; (2) an immersive orientation with the Mount Sinai Hospital specialty inpatient palliative care consultation team, which involved direct shadowing of the specialty palliative care social workers for three months, including joining interdisciplinary team (IDT) rounds and participating in goals-of-care discussions with patients and/or family members; and (3) participation in an intensive two-day *Geritalk*^14,15^ course, an advanced communication skills training course based on *Oncotalk*.^16^

##### Embedding the primary palliative care social worker into the hospital medicine service

After the social worker's palliative care training and orientation were completed, the social worker collaborated closely with the specialty palliative care team through formal and informal meetings. For formal and informal administrative oversight, the social worker had monthly meetings with the Vice-Chair of Inpatient Geriatrics and Palliative Care (EC) and the Director of Quality and Clinical Information (LG) regarding workflow, troubleshooting referrals, and case-based clinical reviews. The social worker also participated in the monthly Hospice and Palliative Care Committee led by the Vice President for Quality Initiatives for the Mount Sinai Hospital and reported the progress of the program's development. In addition, she participated in the weekly palliative care social work clinical meetings with the specialty palliative care social workers, and the specialty palliative care IDT biweekly case-based roundtable discussions. Finally, the social worker attended the monthly hospital medicine staff meetings during which she introduced the program and subsequently presented programmatic updates on a quarterly basis.

To ensure appropriate referrals, the social worker communicated with the specialty palliative care triage nurse each morning, following specialty palliative care IDT rounds; at this point in the morning, the triage nurse already determined which new consultations had no physical symptom management needs, as physical symptom management remained outside of the social worker's clinical scope of practice. Many of these patients came from the specialty palliative care clinical oncology trigger program,^17^ for which patients received an automatic palliative care consult if they had an advanced solid tumor and one of the following: a prior hospitalization within 30 days; hospitalization >7 days; or active symptoms. To promote direct hospital medicine referrals, the social worker also contacted the hospital medicine attendings on service via e-mail, text messaging, or in-person meetings, as well as unit social workers to identify other potential referrals on a daily basis.

##### Developing the framework for social worker-led primary palliative care consultation

**FIG. 2.**
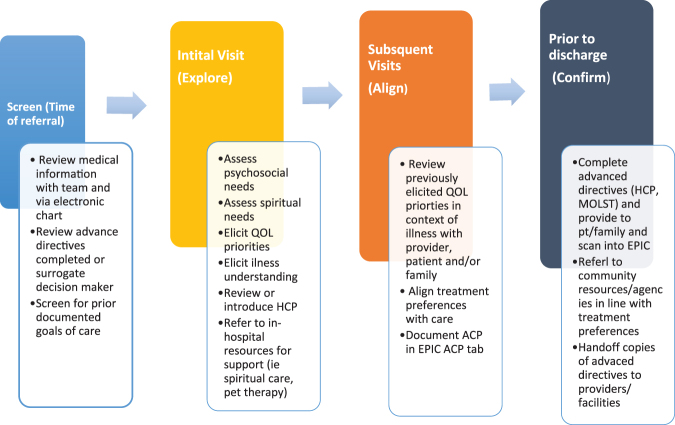
Hospital medicine primary palliative care consultation workflow. This figure provides an overview of the workflow for the Hospital Medicine Primary Palliative Consultation from time of referral to end of the consultation or discharge. ACP, advance care planning; EMR, electronic medical record; QOL, quality of life; MOLST, Medical Orders for Life-Sustaining Treatment.

The framework used to evaluate referred patients focused on three components: exploration, alignment, and finally, confirmation (Fig. 2). This framework describes the social worker's role in the consultations, which included daily communication with the hospital medicine team. Case examples of patients seen by the primary palliative care social worker are found in Table 2.

**Table 2.** Case Examples of Typical Consults Seen by Hospital Medicine Primary Palliative Care Social Worker

Case 1	
Medical background	30-Year-old Cantonese-speaking female with metastatic cervical cancer, admitted for nausea, abdominal pain, and decreased oral intake
Explore	Moved to the United States from China; divorced; family live in China and are unaware of cancer; hoped to be a mother; feels uncertain of future and hoped for answers
Align	Goals-of-care meeting held; prognosis shared with patient and family (over the phone in China); completed advance directives: HCP, DNR/DNI order
Confirm	Discharged with home hospice; mother came to the United States on a visa from China to be by her side until she died
Case 2	
Medical background	74-Year-old male with coronary artery disease, heart failure, and diabetes mellitus admitted with a gastrointestinal (GI) bleed requiring intubation in the surgical intensive care unit, with subsequent extubation and transfer to hospital medicine
Explore	Catholic; no family support/surrogates; values being physically active, cooking, and eating different dishes
Align	GOC meeting held; shared heart failure illness trajectory; advance directives completed: MOLST with trial of critical care
Confirm	Discharged to subacute rehabilitation; completed advance directives and provided to both patient and facility
Case 3	
Medical background	92-Year-old female with advanced dementia admitted for aspiration pneumonia and poor nutritional intake.
Explore	Two daughters identified as surrogate decision makers; Holocaust survivor; had been caregiver for others; value independence; loves animals; would not want to be dependent on “machines”
Align	GOC meeting held; shared trajectory of dementia; completed advance directives: MOLST with DNR/DNI order, no artificial nutrition, antibiotics as needed, and hospice when timing appropriate
Confirm	Discharged home with home care; hospice information and agency options for the future provided; connected to Holocaust survivor supportive agency; referred to pet therapy

This table shows case examples of patients seen by the Hospital Medicine Primary Palliative Care service, including a medical history and the stepwise nature of each consultation including explore, align, and confirm.

DNR/DNI, do not resuscitate/do not intubate; GOC, goals of care; HCP, healthcare proxy; MOLST, Medical Orders for Life-Sustaining Treatment.

#### Exploration: Initial assessment

Before the initial consultation, the primary palliative care social worker collaborated with the hospital medicine team to gain a background understanding of the patient's illness trajectory and prognosis. Next, the primary palliative care social worker conducted a patient assessment, which included (1) checking for understanding of illness, (2) exploration of coping mechanisms including religious and/or cultural values, (3) delivery of psychoeducation on ACP, and (4) eliciting QOL priorities in the context of illness and hopes for treatment.

After the initial assessment, the primary palliative care social worker provided a summary of her assessment and recommendations to the hospital medicine team. For example, based on her initial assessment, she would recommend having joint goals-of-care discussion with the patient, surrogate medical decision-makers, and hospital medicine team, to deliver serious news and discuss next steps in care plan. Depending on the anticipated content of the meeting, the social worker teamed up with members of the primary team (e.g., the hospital medicine attending or house staff, depending on availability) to provide medical updates. Following the meeting, she would recommend completion of relevant documents, including advance directives, do-not-resuscitate documents, or Medical Orders for Life-Sustaining Treatment (MOLST) forms. The social worker also referred patients to any inpatient hospital resources that might aid in the patients' comfort and coping, such as spiritual care, pet therapy, art therapy, music therapy, and child life.

#### Alignment: Follow-up visit

The first follow-up visit focused on facilitating an ACP and/or goals-of-care discussion based upon the values elicited during the initial assessment. These interdisciplinary meetings helped align the patient's values to the existing treatment options. The participants typically included the patients and/or their surrogate decision-maker, a hospital medicine physician, the primary palliative care social worker, and the inpatient or outpatient clinicians pertinent to the patient's care, such as specialty physicians, social workers, and chaplains. After values were aligned with treatment decisions, relevant advance directive documents were completed, based on patient or surrogate willingness, to reflect those values and plan for the future. Documentation of patient wishes was also recorded by the primary palliative care social worker and hospital medicine teams in the “Advanced Care Planning” tab of the patient's electronic medical record (EMR), the location in the EMR where copies of completed relevant advance directive forms are stored and goals-of-care discussions held between patients, their surrogates, and interdisciplinary team members are documented. This ensures that patients' values, goals, and treatment preferences are easily accessible for clinicians throughout the health system.

#### Confirmation: Closing visit(s)

Closing visit(s), completed by the primary palliative care social worker, centered on the confirmation of the patient's understanding of the treatment plan, as well as grief and anticipatory bereavement counseling for difficult emotions evoked by the goals-of-care discussion. In addition, the confirmation visits incorporated collaboration with the interdisciplinary team to establish a discharge plan that aligned with the patient's goals and ensure the patient received appropriate discharge resources, such as support groups and psychotherapy resources. Finally, the primary palliative care social worker provided a “warm” hand-off via phone call or e-mail about the established care plan to the clinician(s) assuming the patient's postdischarge care. For example, she contacted the outpatient primary care clinician(s) or facility-based clinician(s) from subacute rehabilitation, nursing homes, or dialysis centers, and specifically provided them with a summary of the goals-of-care discussions and a copy of the completed advance directives.

### Data collection and analysis

To evaluate the program, we collected data about the clinical information (primary diagnosis, Karnofsky Performance Status Index^18^), primary palliative care utilization (ability to participate in goals-of-care discussion, referral source, documented ACP discussions, specialty palliative care referrals), and discharge disposition (home, facility, or hospice, or in-hospital death). We obtained hospital-level demographic and health care utilization data from the Mount Sinai Hospital databases (including age, gender, race, insurance, hospital length of stay, intensive care unit utilization, and 30-day readmission). Univariate analyses were conducted using Stata, version 16.1. We obtained institutional review board approval for this project.

## Results

### Demographics

In the program's first year, the hospitalist primary palliative care social worker saw 232 patients: average age of 69 years, 104 (44.8%) female and 79 (34.1%) white (Table 1). The population seen had a median Karnofsky Performance Status of 40%, meaning they were “disabled and required special care and assistance.” In addition, 159 (68.5%) of patients seen by the program had decision-making capacity to actively participate in goals-of-care discussion. The most common primary diagnoses were as follows: 134 (57.76%) cancer, 42 (18.1%) heart failure, 47 (20.3%) dementia, 35 (15.09%) chronic obstructive pulmonary disease, 10 (4%) liver disease, and 8 (3.5%) stroke. The mean hospital length of stay was 16.5 days (median of 10 days). Of those seen, 85 (37%) had Medicare, 63 (27.2%) Medicare HMO, 34 (14.7%) Medicaid HMO, 12 (5.2%) PPO, and 9 (4%) Medicaid.

**Table 1.** Demographics and Clinical Characteristics of Patients Admitted to Hospital Medicine Service Who Received Social Worker-Led Primary Palliative Care Consultation (*N* = 232)

	N (%)
Mean age, years	69 years
Female	104 (45)
Race	
White	79 (34)
African American	42 (18)
Asian	17 (7)
Other	80 (35)
Unknown	14 (6)
Referral	
Oncology	113 (49)
Primary	42 (18)
Attending	34 (15)
Sepsis	24 (10)
Other (referral source)	19 (8)
Diagnoses	
Cancer	134 (58)
Dementia	47 (20)
Heart failure	42 (18)
Chronic obstructive pulmonary disease (COPD)	35 (15)
Hospitalized in prior six months	94 (41)
Karnofsky Performance Status, median	40%
Capacity to participate in goals of care	159 (68.5)

This table reports descriptive results of patients seen by the Hospital Medicine Primary Palliative Care Service.

### Hospitalist primary palliative care service delivery pattern

About half of patients were referred from the specialty palliative care solid tumor oncology trigger program,^17^ 42 (18%) from the inpatient specialty palliative care consultation service, and 34 (14.7%) were referred directly by hospital medicine. Patients were seen by the primary palliative care social worker a median of three days (mean of 6.32 days) after admission and followed by the social worker for a median of two days (mean of three days). At the time of referral, 159 (68.5%) patients were able to participate in discussion (Table 1).

After the palliative care social worker consultation, 207 (89%) patients had goals-of-care conversation documented in our EMR, compared with 10 (4.3%) before the consultation. About one-quarter of patients (*N* = 60) were subsequently seen by the specialty palliative care inpatient consultation team for either transfer to the palliative care unit (31 [51.6%]) or for management of complex physical symptoms (29 [48.3%]).

### Health care utilization and discharge disposition

**FIG. 3.**
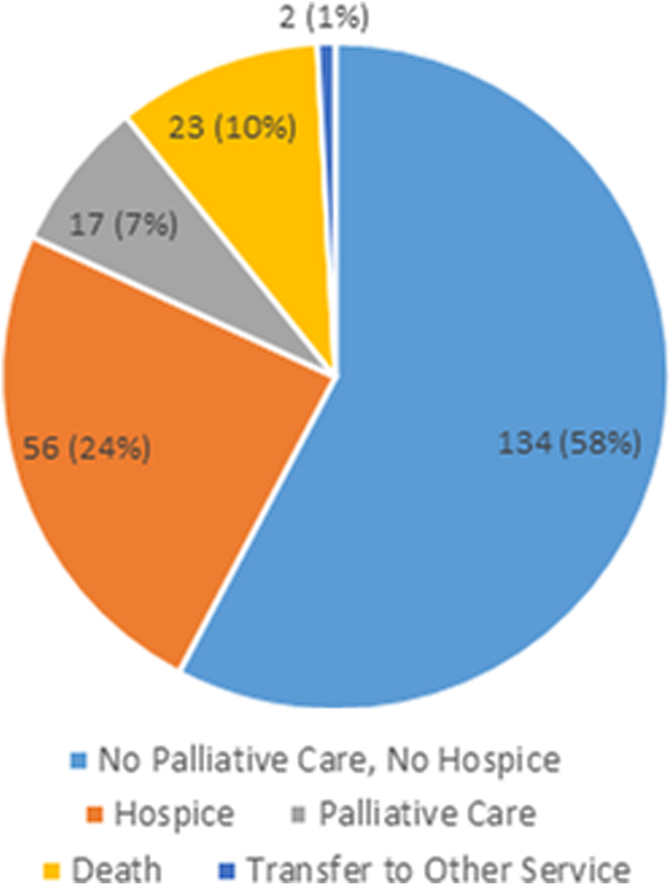
Discharge disposition of patients seen by Hospital Medicine social worker-led primary palliative care. This figure depicts that setting of discharge for patients seen by the Social Worker-Led Hospital Medicine Primary Palliative Care Service.

About 13% (*n* = 29) of patients were admitted to the intensive care unit during the hospitalization, of which 22 (76%) occurred before the primary palliative care consultation and 7 (24%) occurred after the consultation. On discharge, 56 (24.1%) patients were discharged with hospice (home hospice: 22 [39.3%] and facility-based hospice 34 [60.7%]). Overall, 17 (7.3%) were discharged with community-based palliative care services (Fig. 3). Overall, 23 (10%) patients died during the hospitalization. The 30-day readmission rate for those seen by the primary palliative care social worker was 22 (9.5%) with a median of 11 hospital-free days.

## Discussion

The hospital medicine social worker-led primary palliative care model aimed to improve primary palliative care service delivery to patients admitted to the hospital medicine service. The model demonstrates it is feasible to elicit patient values through the primary palliative care social work assessment and facilitate goals-of-care discussions based upon those elicited values. As a result, the program improved patient-directed goals-of-care documentation and maintained a comparable hospice referral rate with that of specialty palliative care among patients earlier in the disease trajectory.

Important to note is that a majority of those seen by the social worker-led primary palliative care program were seen earlier in the disease course, as supported by the higher Karnofsky Performance Status Index (less functional impairment, better survival),^19^ and ability to participate in goals-of-care discussions regarding future medical care. By identifying patients during this key window, they are able to share directly their life values and the impact of disease on their QOL. Ensuring that patients are able to participate in their own goals-of-care discussions is important because studies suggest that surrogates are not able to identify accurately the preferences of their loved ones.^20^ This upstream model also allowed the primary palliative care social worker to explore differences in opinions about goals-of-care between patients and their families, to facilitate communication of patients' wishes, and finally to help rectify those differences before the patient experiences a clinical decline.^21^ By discussing goals of care directly with patients, and not surrogates, in advance of a clinical decline, patients could explore, reflect, and process their illness, and in turn prepare for the inevitable while having choice and control in that process. Due to the complexity and challenges of medical decision making and ACP, these upstream conversations do not negate the need for a conversation about goals and values at the time of a change in clinical condition.

### Limitations

This program evaluation was a retrospective cohort study at a single site. Although we do not have a severity of illness index to compare hospital medicine patients seen by the primary palliative care program and those not seen, the 30-day readmission rate for those seen by the primary palliative care social work-led program was half (9.48%) of that of the overall hospital medicine readmission rate (18%). This finding likely underestimates the readmission rate difference because patients referred to the primary palliative care program likely had a higher illness severity. In addition, with a social worker working alone, this program was able to generate a comparable hospice referral rate (24.1%) with that of the solid tumor specialty palliative care trigger program (26%) at the same hospital,^17^ which comprised a team of at least three disciplines. Finally, the primary palliative care social worker was unable to address physical symptom management needs of these patients, and therefore referred 13% of patients to specialty palliative care for symptom management. To address this gap, the program expanded to a joint primary palliative care social worker and nurse practitioner team. We did not collect data about the willingness to discuss goals of care or clinical circumstance that did or did not warrant a goals-of-care discussion before the time of the consultation. Furthermore, we did not collect information about cost of the program. Future work is needed to examine cost effectiveness of the program, as well as patient-reported outcomes including satisfaction with communication and clinician assessments including burnout.

## Conclusions

We developed an innovative social worker-led primary palliative care program to increase palliative care access to hospital medicine patients in the setting of specialty palliative care workforce shortages. This primary palliative care social worker-led model allowed patients to explore life values and how these values affect treatment decisions and goals of care. These conversations earlier in their disease trajectory allowed the patient's voice to be heard and communicated to their family and care providers, while also allowing patients and family to process anticipatory grief and existential distress with necessary support. This social worker-led primary palliative care program was feasible, expanded the reach of palliative care, increased goals-of-care documentation, and maintained a hospice referral rate comparable with a specialty palliative care inpatient consultation service. Further research is needed to develop and test models of palliative care that increase access to high-quality palliative care for patients with serious illness while also preserving the limited resource of specialty palliative care for those with the most complex needs.

## Abbreviations Used

ACP: advance care planning
DNR/DNI: do not resuscitate/do not intubate
EMR: electronic medical record
GOC: goals of care
IDT: interdisciplinary team
HMO: health maintenance organization
MOLST: Medical Orders for Life-Sustaining Treatment
PPO: preferred provider organization
QOL: quality of life
